# HDAC6 inhibition reduces *Pseudomonas aeruginosa* adherence and internalization in cystic fibrosis epithelial cells via microtubule stabilization

**DOI:** 10.1128/iai.00225-25

**Published:** 2026-08-12

**Authors:** Kathleen L. Boyne, Conor Broeking, Mark Blackstone, Sruthi Singaraju, Thomas J. Kelley, Deborah A. Corey, Patrick C. Seed

**Affiliations:** 1 Department of Pediatrics, Division of Pulmonary and Sleep Medicine, Ann & Robert H. Lurie Children's Hospital of Chicago, Northwestern University, Feinberg School of Medicine 3270, Chicago, Illinois, USA; 2 Stanley Manne Children’s Research Institute, Ann & Robert H. Lurie Children’s Hospital of Chicago, Chicago, Illinois, USA; 3 Department of Genetics and Genome Sciences, Case Western Reserve University 2546, Cleveland, Ohio, USA; 4 Department of Pediatrics, Division of Infectious Diseases, Ann & Robert H. Lurie Children's Hospital of Chicago, Northwestern University, Feinberg School of Medicine 3270, Chicago, Illinois, USA; University of California Davis, Davis, California, USA

**Keywords:** histone deacetylase 6, microtubules, airway epithelium, *Pseudomonas aeruginosa*, cystic fibrosis

## Abstract

*Pseudomonas aeruginosa* is a common opportunistic pathogen that causes chronic lung infections in individuals with cystic fibrosis. Despite advances in therapies that restore cystic fibrosis transmembrane conductance regulator function, persistent colonization of the airway remains a major clinical challenge. Reduced clearance of *P. aeruginosa* from the cystic fibrosis airway has been associated with the increased activity of histone deacetylase 6 (HDAC6), a cytoplasmic deacetylase that decreases microtubule acetylation and stability. In this study, we investigated the role of HDAC6 in modulating interactions between *P. aeruginosa* and cystic fibrosis airway epithelial cells. Pharmacologic inhibition of HDAC6 significantly reduced bacterial adherence in both mouse and human cystic fibrosis epithelial cells. Genetic deletion of HDAC6 produced similar effects, while knockout of a microtubule-stabilizing protein increased bacterial adherence, mimicking the cystic fibrosis phenotype. HDAC6 inhibition also reduced bacterial internalization, although to a lesser extent compared to adherence. These results suggest that microtubule destabilization contributes to the enhanced colonization of cystic fibrosis airways by *P. aeruginosa*. Targeting host microtubule regulatory pathways, particularly by inhibiting HDAC6, may represent a promising host-directed strategy to limit early bacterial attachment and reduce the risk of chronic infection in cystic fibrosis.

## INTRODUCTION

Cystic fibrosis (CF) is a genetic disorder characterized by chronic airway infections, inflammation, and impaired mucociliary clearance, largely due to defective cystic fibrosis transmembrane conductance regulator (CFTR) function. CFTR is a chloride and bicarbonate channel important for maintaining water and electrolyte balance in the airway. Mutations in the CFTR gene impair this function and lead to thickened mucus, providing a conducive environment for bacterial colonization in the CF airway. However, even with recent advances in treatments, including CFTR modulator therapies that appear to reduce airway mucus burden, many patients fail to clear pathogens such as *Pseudomonas aeruginosa* (PA) (1–4). This suggests that impaired mucus clearance alone does not fully account for the susceptibility of CF airways to colonization, and that additional cellular mechanisms governing interactions between airway cells and bacterial pathogens warrant continued investigation (5–21). Recent studies have implicated histone deacetylase 6 (HDAC6) in this process, demonstrating that HDAC6 inhibition or depletion improves PA clearance in CF models, although the epithelial mechanisms underlying this effect remain unclear (22, 23).

HDAC6 is a cytoplasmic deacetylase that targets α-tubulin, a key structural component of microtubules, thereby playing a central role in regulating microtubule dynamics (24–26). Microtubule dynamics are known to be impaired in CF epithelial cells, where increased HDAC6-dependent deacetylase activity contributes to decreased α-tubulin acetylation and reduced microtubule stability (27–29). Microtubules provide tracks for vesicular transport and aid in the polarized delivery and retrieval of membrane proteins in epithelial cells (30). As such, their dysregulation could alter the airway epithelial surface encountered by bacteria or affect pathways involved in bacterial uptake. However, whether impaired microtubule regulation directly alters PA adherence or internalization in CF airway epithelial cells has not been previously established.

Epithelial polarity and membrane organization are important determinants of PA interactions with host cells (8, 10, 15, 16). For example, Bucior et al. demonstrated that PA targets distinct epithelial receptors based on cell polarity (17). Although this study did not examine microtubule dysfunction or CF-specific receptor trafficking, it highlights the importance of epithelial surface organization in host-PA interactions and provides additional rationale for investigating whether impaired microtubule regulation influences bacterial adherence and internalization. Recent studies have also linked dysregulation of additional microtubule-associated proteins, such as tubulin polymerization-promoting protein (TPPP) loss, to CF-like airway responses to infection, including the impaired clearance of PA in airways (31). TPPP loss or dysfunction is expected to reduce microtubule polymerization and stability.

Our study builds on these findings by investigating the hypothesis that HDAC6-driven microtubule instability contributes to increased PA adherence and internalization in CF epithelial cells. By utilizing both mouse nasal epithelial (MNE) cells and human nasal epithelial (HNE) cells and human cell models, we demonstrate that pharmacologic HDAC6 inhibition with tubastatin, which increases microtubule acetylation and stability, significantly reduces bacterial adherence and internalization in CF cells, bringing these parameters closer to those observed in non-CF cells. Furthermore, we confirm these findings in genetic knockout (KO) mouse models, providing a potential epithelial mechanism for the improved bacterial clearance previously observed following HDAC6 inhibition or depletion. Our findings provide novel insights into the molecular underpinnings of bacterial colonization in CF airways and suggest that therapeutic targeting of microtubule stability could offer a new strategy for limiting colonization of bacterial pathogens in patients with CF.

## RESULTS

### HDAC6 inhibition reduced PA adherence in CF airway epithelial cells

**Fig 1:**
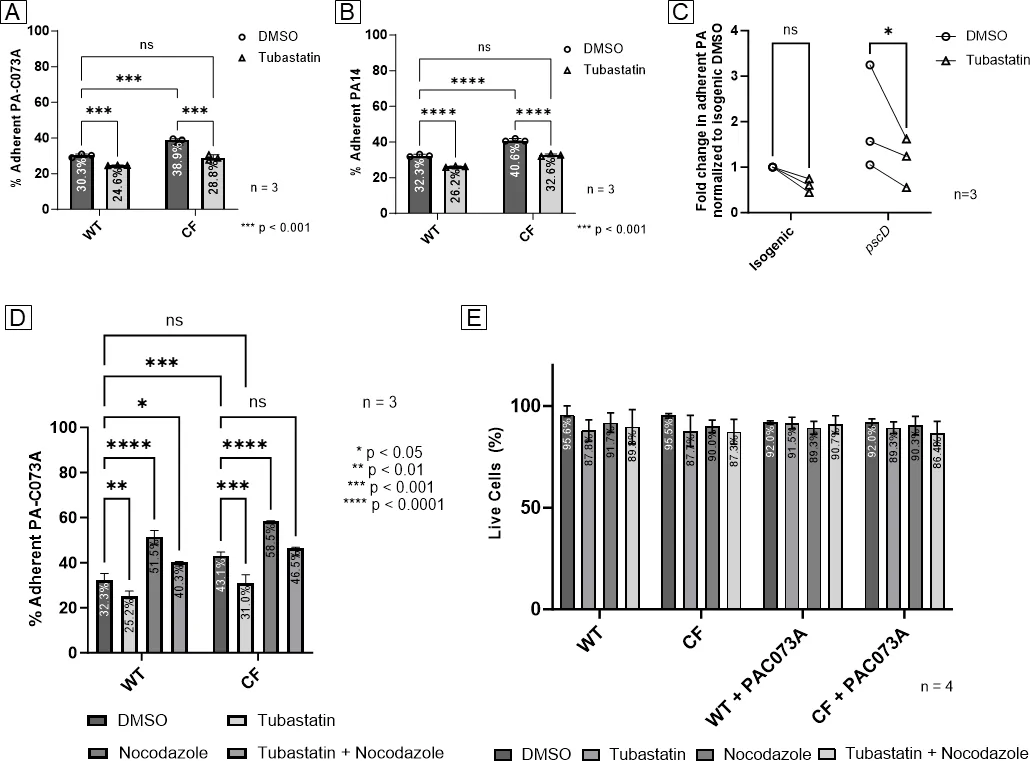
HDAC6 inhibition reduces PA adherence in CF epithelial cells via microtubules. WT and CF mouse or human nasal epithelial cells were grown at ALI, treated with tubastatin (10 µM), nocodazole (40 µM), and/or vehicle (DMSO), then apically infected with (A) clinical CF PA isolate, PA-C073A, (**B**) laboratory strain, PA14, or (C) T3SS-deficient PA strain, *pscD*, at a multiplicity of infection of 1:30 for 30 min. Adherence is represented as the percentage of total bacteria adhered or as fold change. (**A**) Adherence of CF clinical isolate PA-C073A was increased in CF MNE cells relative to WT. This was effectively normalized in CF to WT levels by treatment with an HDAC6-selective inhibitor and microtubule-stabilizing agent, tubastatin. (**B**) Adherence of PA laboratory strain PA14 was similarly increased in CF MNE cells relative to WT cells, with reversal of this phenotype by treatment with tubastatin. Results are displayed as median ± interquartile range. Significance was determined by two-way ANOVA with *post hoc* Tukey’s multiple comparisons test. *n* = 3 biological replicates, with technical replication in triplicate. (**C**) Tubastatin reduced adherence of both the isogenic control and *pscD* mutant in CF HNEs, reaching statistical significance in the *pscD* mutant group. This suggests that the effect of HDAC6 inhibition on PA adherence is not solely dependent on an intact T3SS (*P < 0.05). Data shown as fold change relative to isogenic DMSO, with paired points representing matched independent experiments. Significance was determined by two-way ANOVA with planned comparisons; *P* values not adjusted. (**D**) Microtubule destabilizing agent, nocodazole, resulted in increased adherence of PA-C073A in WT and CF MNE cells, and substantially counteracted the effects of tubastatin, suggesting tubastatin is acting via microtubule stabilization. Significance was determined as above (**A** and **B**). (**E**) Microtubule-targeted agents impacted WT and CF cell viability similarly without leading to significant differences in cell death. After 30 min of incubation with PA-C073A or no bacteria, cells were detached with trypsin. An equal volume of trypan blue was added to a 20 µL aliquot of each cell suspension, and live vs dead cells were counted using the Countess 3. Degree of cytotoxicity is presented as the percentage of live cells following treatment. No statistically significant differences were observed across experimental conditions. *n* = 4 biological replicates, with technical replication in duplicate. Significance was determined by two-way ANOVA with *post hoc* Tukey’s multiple comparisons test. ALI, air-liquid interface; CF, cystic fibrosis; DMSO, dimethyl sulfoxide; HDAC6, histone deacetylase 6; HNE, human nasal epithelial; MNE, mouse nasal epithelial; PA, *Pseudomonas aeruginosa*; T3SS, type 3 secretion system; WT, wild type.

To evaluate the impact of HDAC6 inhibition on PA adherence, we compared the adherence of two strains of PA to CF (F508del/F508del) and wild-type (WT) MNE cells grown at the air-liquid interface (ALI) (23, 27). Our results demonstrated significantly increased adherence of the clinical CF isolate PA-C073A to CF MNE cells (38.9% of inoculum) compared to WT MNE cells (30.3%) (Fig. 1A). Strain PA14 adhered similarly, where adherence was higher in CF MNE cells (40.6% of inoculum) relative to WT MNE cells (32.3%) (Fig. 1B).

Treatment with the HDAC6-selective inhibitor tubastatin, which enhances microtubule acetylation and stability, significantly reduced bacterial adherence in both WT and CF MNE cells (Fig. 1A and B). Specifically, tubastatin decreased the adherence of PA-C073A to WT MNE cells from 30.3% of the inoculum to 24.6% and to CF MNE cells from 38.9% to 28.8% (Fig. 1A). Similarly, for PA14, adherence was reduced from 32.3% of the inoculum to 26.2% in WT MNE cells and from 40.6% to 32.6% in CF MNE cells following tubastatin treatment (Fig. 1B).

Given that PA’s type 3 secretion system (T3SS) can facilitate the translocation of virulent effector proteins (such as ExoS and ExoT) into host cells where they can modulate cytoskeletal dynamics, we performed complementary experiments to determine if the observed effect of HDAC6 inhibition on PA adherence required an intact T3SS. We compared the effect of tubastatin on adherence of an isogenic parental strain of PA14 and a *pscD-*deficient mutant without a functional T3SS apparatus to primary HNE cells (32). Tubastatin significantly reduced adherent PA in all paired experiments in cells inoculated with the *pscD* mutant strain (Fig. 1C; *P* = 0.04). These findings suggest the observed reduction in adherence following tubastatin treatment was not mediated solely through T3SS-dependent mechanisms.

The specific involvement of microtubules in bacterial adherence was further supported by experiments using the microtubule disruptor nocodazole. Inhibiting microtubule polymerization with nocodazole increased bacterial adherence in both WT and CF MNE cells, and this effect was strong enough to counteract the reduction in adherence induced by tubastatin treatment (Fig. 1D). Average adherent and total recovered colony-forming units per well are shown for all experiments in Tables S1 to S11. Cell viability in the MNE cells following treatment with tubastatin and nocodazole was similar in WT and CF cells, suggesting that cell death over the assay time course did not contribute to the differential adherence levels (Fig. 1E).

### HDAC6 and TPPP modulated bacterial adherence in mouse nasal epithelial cells

**Fig 2:**
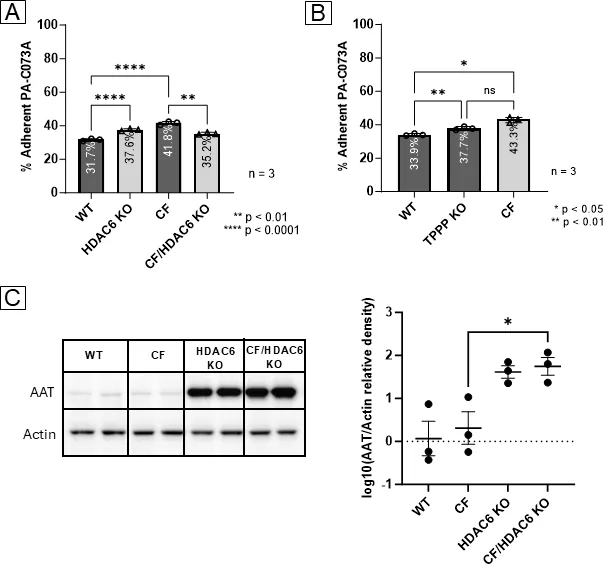
Impact of HDAC6 and TPPP knockout on bacterial adherence in MNE Cells. MNE cells were grown at ALI, then apically infected with a clinical CF isolate of *Pseudomonas aeruginosa*, PA-C073A, at a multiplicity of infection of 1:30 for 30 min. Adherence is represented as the percentage of total bacteria adhered. (**A**) Bacterial adherence of PA-C073A was increased in CF MNE cells relative to WT; however, HDAC6 depletion in CF mice reduced the degree of bacterial adherence. HDAC6 depletion alone in WT mice resulted in a slight increase in adherence and may be attributable to increased inflammation observed in prior studies. (**B**) Bacterial adherence of PA-C073A to TPPP KO MNE cells was increased relative to WT cells and was similar to that observed in CF MNE cells. Data are presented as median ± interquartile range. Statistical significance was determined by two-way ANOVA with *post hoc* Tukey’s multiple comparisons test. *n* = 3 biological replicates, with technical replication in triplicate. (**C**) MNE cells were grown at ALI, lysed, and analyzed for acetylated α-tubulin (AAT) abundance via western blot. Representative blots are shown (left) and quantified by densitometry (right). Band density was normalized to actin and log10-transformed prior to analysis to account for the wide range in band density. Data are shown as mean ± SEM. Each point represents an independent experiment (*n* = 3), with technical duplicates averaged within each experiment. Statistical significance was determined by repeated-measures one-way ANOVA with Tukey’s multiple comparisons test. AAT, acetylated α-tubulin; ALI, air-liquid interface; CF, cystic fibrosis; HDAC6, histone deacetylase 6; KO, knockout; MNE, mouse nasal epithelial; PA, *Pseudomonas aeruginosa*; SEM, standard error of the mean; TPPP, tubulin polymerization-promoting protein; WT, wild type.

To further elucidate the role of microtubule stability in bacterial adherence, we investigated the effects of genetic knockout of HDAC6 and TPPP in primary MNE cells. MNE cells from previously validated wild-type (WT, C57BL/6J, B6), cystic fibrosis (CF, *Cftr^F508del^*^/*F508del*^), HDAC6 null (HDAC, *Hdac6*^−/−^), CF deficient of HDAC6 (CF/HDAC, *Cftr^F508del^*^/*F508del*^/*Hdac6*^−/−^), and TPPP null (TPP, *Tppp*^−/−^) mice were harvested and grown at ALI (23, 27, 31). HDAC6 KO in CF mice significantly decreased bacterial adherence from 41.8% of the inoculum in CF MNE cells to 35.2% in CF/HDAC6 KO MNE cells (Fig. 2A). Interestingly, HDAC6 KO alone led to a modest increase in bacterial adherence compared to WT controls (Fig. 2A).

Conversely, TPPP knockout, which reduces microtubule stability, resulted in significantly higher bacterial adherence compared with WT MNE cells. Specifically, bacterial adherence increased from 33.9% of the inoculum in WT MNE cells to 37.7% in TPPP KO MNE cells. This was similar to the adherence observed in CF MNE cells, which showed that 43.3% of the PA-C073A inoculum adhered to the apical cell surface (Fig. 2B).

Given that HDAC6 inhibition is expected to increase α-tubulin acetylation, we confirmed the effect of HDAC6 loss on acetylated α-tubulin abundance in MNE cells grown at ALI. Western blot analysis demonstrated increased acetylated α-tubulin in HDAC6 KO and CF/HDAC6 KO MNE cells compared with WT and CF controls, with a significant increase in CF/HDAC6 KO MNE cells compared with CF MNE cells (Fig. 2C; *P* < 0.05). These findings support that HDAC6 loss increases microtubule acetylation in this model.

### HDAC6 inhibition reduced bacterial internalization in CF epithelial cells

**Fig 3:**
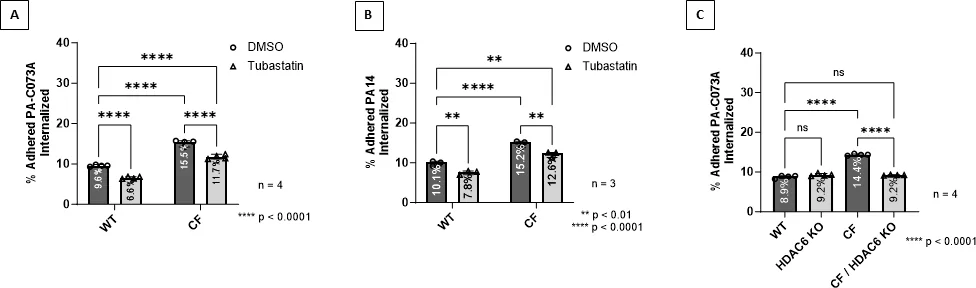
HDAC6 inhibition partially reduces bacterial internalization in CF MNE cells. MNE cells were grown at ALI, apically infected with clinical CF isolate of *Pseudomonas aeruginosa*, PA-C073A, or laboratory strain, PA14, at a multiplicity of infection of 1:30 for 2 h, then treated with gentamicin to kill adhered but not internalized bacteria. Internalization is represented as the percentage of adherent bacteria that were internalized. Internalization of PA-C073A (**A**) and PA14 (**B**) was more than 1.5 times greater in CF compared to WT MNE cells. Tubastatin resulted in a significant decrease in internalization in WT and CF cells, though not quite back to that of WT levels in CF. (**C**) Depletion of HDAC6 in CF mice (CF/HDAC6 KO) resulted in a significant decrease in internalization of PA-C073A, comparable with WT and HDAC6 KO levels. Data are presented as median ± interquartile range. Statistical significance was determined using two-way ANOVA with *post hoc* Tukey’s multiple comparisons test. *n* = 4, *n* = 3, and *n* = 4 biological replicates, respectively, with technical replication in duplicate. ALI, air-liquid interface; CF, cystic fibrosis; HDAC6, histone deacetylase 6; KO, knockout; MNE, mouse nasal epithelial; PA, *Pseudomonas aeruginosa*; WT, wild type.

We next assessed whether microtubule stabilization affected PA internalization in CF and WT epithelial cells. CF MNE cells exhibited significantly increased internalization of PA-C073A (15.5% of adhered bacteria) compared to WT MNE cells (9.6%; *P* < 0.0001) (Fig. 3A). Similarly, PA14 internalization was higher in CF MNE cells (15.2% of adhered bacteria) than in WT MNE cells (10.1%) (Fig. 3B).

Treatment with tubastatin led to a partial reduction in bacterial internalization in CF MNE cells. Internalization of PA-C073A decreased from 15.5% of adhered bacteria to 11.7% with tubastatin treatment, though this did not fully restore WT levels (Fig. 3A). Similar trends were observed for PA14, where internalization decreased from 15.2% to 12.6% following tubastatin treatment (Fig. 3B). In the HDAC6 KO mice, internalization of PA-C073A was significantly reduced in CF/HDAC6 KO MNE cells compared to CF MNE cells, with levels comparable to those in WT MNE cells (Fig. 3C).

### Human cell models confirmed findings from mouse models

**Fig 4:**
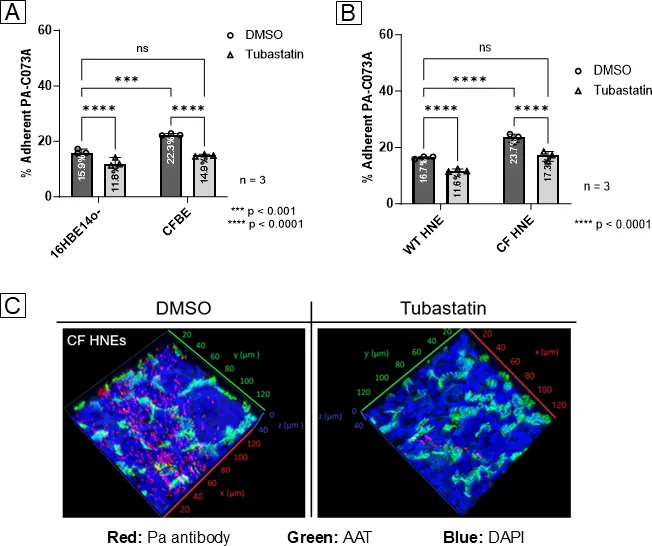
Increased bacterial adherence in human cell models of cystic fibrosis is reversed by microtubule stabilization with tubastatin. Cells were grown at ALI, treated with tubastatin or vehicle for 24 h, then apically infected with clinical CF isolate, PA-C073A, at a multiplicity of infection of 1:30 for 30 min. Adherence is represented as the percentage of total bacteria adhered. (**A**) Bacterial adherence of PA-C073A was increased in CFBE (CF) cells relative to 16HBE14o^−^ (non-CF) cells. Tubastatin treatment significantly reduced the degree of adherence in CF cells to a level comparable to non-CF cells. (**B**) Bacterial adherence of PA-C073A to CF primary HNE cells was similarly increased at baseline and restored to WT levels with tubastatin treatment. Data are presented as median ± interquartile range. Significance was determined by two-way ANOVA with *post hoc* Tukey’s multiple comparisons test. *n* = 3 biological replicates, with technical replication in triplicate. (**C**) Confocal imaging of PA-C073A (red) and acetylated α-tubulin (AAT; green) stains in CF HNE cells grown at ALI provided further evidence of reduced PA adherence in CF cells following tubastatin treatment. Z-stack images were captured across three trials at 40× to generate the representative 3D images here. AAT, acetylated α-tubulin; ALI, air–liquid interface; CF, cystic fibrosis; CFBE41o^−^, cystic fibrosis bronchial epithelial cell line; HDAC6, histone deacetylase 6; HNE, human nasal epithelial; PA, *Pseudomonas aeruginosa*; WT, wild type; 16HBE14o^−^, non-CF human bronchial epithelial cell line.

To validate our findings in human models, we examined bacterial adherence in human cell lines and primary HNE cells. CFBE41o^−^ cells, which are representative of CF epithelium, exhibited significantly increased adherence of PA-C073A compared to non-CF 16HBE14o^−^ control cells (Fig. 4A). Primary CF HNE cells also showed higher bacterial adherence compared to non-CF HNE cells (Fig. 4B). Consistent with our findings in mouse cells, HDAC6 inhibition with tubastatin significantly reduced bacterial adherence in both CFBEs and CF HNE cells (Fig. 4A and B). Notably, in CF HNE cells, tubastatin treatment restored bacterial adherence to levels observed in non-CF HNE cells. These findings were further confirmed by visualization on confocal microscopy in CF HNE cells (Fig. 4C).

## DISCUSSION

Chronic PA infections are a hallmark of CF and a major driver of disease morbidity. Despite significant improvements in CFTR-targeted therapies, including elexacaftor/tezacaftor/ivacaftor, airway dysbiosis and pathogen susceptibility persist (1–4). Our study further establishes HDAC6 as a critical regulator of this susceptibility, with evidence across murine and human models that HDAC6 inhibition significantly reduces PA adherence to CF airway epithelial cells.

HDAC6 regulates the acetylation state of α-tubulin, a key determinant of microtubule stability. In CF, elevated HDAC6 activity leads to hypoacetylated, destabilized microtubules, disrupting cellular processes that are critical to maintaining epithelial integrity and defense (23, 27, 28, 31, 33, 34). Bacterial adherence to the epithelium represents one of the earliest and most modifiable steps in bacterial colonization. Our findings consistently show that pharmacologic inhibition and genetic depletion of HDAC6 restore adherence in CF cells to levels comparable with non-CF controls. This phenotype was reversed with HDAC6 inhibitor treatment, tubastatin, across multiple cell types—human and murine—including CFBE41o^−^ cells, CF MNE cells, and primary CF HNE cells. In contrast, genetic loss of TPPP, a microtubule-stabilizing protein, increased bacterial adherence in WT MNE cells, mimicking the CF phenotype. These parallel findings support a model in which microtubule instability, driven by HDAC6 activity, impairs the cell’s ability to restrict bacterial binding at the epithelial surface.

These adherence findings were robust, reproducible, and more pronounced than effects on internalization, reinforcing the hypothesis that HDAC6 plays a primary role in early host defense at the epithelial cell surface. We propose that this results from disruptions in pathogen receptor localization/turnover and epithelial polarity given the central role of microtubules in these processes. This is consistent with previously reported findings that PA exploits apical and basolateral receptors differentially depending on epithelial polarity, influencing both bacterial adherence and internalization (17). In CF, chronic inflammation and epithelial injury can disrupt polarity and exacerbate receptor exposure of potential binding sites for PA (8, 11, 13, 35). HDAC6 inhibition may help preserve polarity and epithelial organization, thereby limiting aberrant exposure of these sites in CF. Of note, HDAC6 KO in WT mice resulted in increased adherence, which may be attributable to a relative increase in epithelial injury related to the heightened inflammatory response previously described in these mice in the absence of a CF background (23). This highlights the complex role of HDAC6, where anti-inflammatory and barrier-preserving effects may be particularly beneficial in the context of CF.

Our findings also align with those of Darling et al., who similarly used polarized bronchial epithelial (BE) cells and reported increased internalization (36). In contrast, Kowalski and Pier noted lower rates of bacterial internalization in CF models (37). This discrepancy may stem from methodological differences, including bacterial strain, CFTR reconstitution, and/or variations in cell polarization. Our protocol also used a lower gentamicin dose for a longer duration to minimize host cell toxicity while ensuring complete killing of external bacteria, potentially permitting limited bacterial replication. Jolly et al. similarly observed increased intracellular bacterial persistence in CF (38). Previous studies also suggest that bacterial internalization in CF cells may involve additional factors such as dysregulated endocytic pathways and perturbations in cAMP-dependent signaling that warrant further investigation. These findings suggest that internalization outcomes are context-dependent and influenced by bacterial and host factors. Nonetheless, our data support the conclusion that HDAC6 regulates early epithelial interactions with PA, with adherence being the most consistent and modifiable outcome. This reinforces the *in vivo* finding of improved clearance of PA observed in CF mice deficient of HDAC6 (CF/HDAC, *Cftr*^F508del/F508del^/*Hdac6*^−/−^) by Rosenjack et al. and provides a potential mechanism for this observed improvement (23).

Importantly, PA itself can alter host-cell architecture through several virulence pathways, including T3SS effectors that target host cytoskeletal elements. For example, PA toxins can destabilize microtubules by hyperphosphorylating tau, impairing endothelial barrier function (39). Our study suggests that enhancing microtubule stability by inhibiting HDAC6 can reverse this pathogenic advantage and strengthen epithelial defenses. To determine whether the effect of HDAC6 inhibition on adherence required this bacterial pathway, we examined a *pscD*-deficient mutant lacking a functional T3SS apparatus (32). Tubastatin continued to reduce adherence, suggesting the observed effect was not solely dependent on T3SS-mediated mechanisms and supporting an important host-cell contribution. PA can also establish persistent, biofilm-like infections, resulting in chronic inflammation and epithelial dysfunction as seen in ALI models of CF epithelium (40). By reducing initial adherence, our results suggest that HDAC6 inhibition may limit the initial colonization events that precede biofilm formation, potentially mitigating the burden of chronic infection. HDAC6 inhibition may also affect PA directly, as Barone et al. demonstrated in a study showing a reduction in swarming motility and biofilm formation following direct exposure of PA to a selective HDAC6 inhibitor (41). Together, these findings support the possibility that HDAC6-targeted therapy may provide benefit through complementary effects on both the airway epithelium and the pathogen.

Overall, while our data strongly support HDAC6 as a promising therapeutic target for reducing PA interactions with CF airway epithelial cells, this study is limited by its reliance on *in vitro* and *ex vivo* models. Although prior work demonstrated improved bacterial clearance in *Cftr*^F508del/F508del^/*Hdac6*^−/−^ mice *in vivo*, future short-term intravital imaging studies, complemented by high-resolution *ex vivo* airway imaging, could help define the early events underlying this benefit by assessing the localization of fluorescently labeled PA, epithelial surface organization, candidate receptor distribution, and interactions with recruited phagocytes immediately following inoculation. Such studies could also directly evaluate whether HDAC6 inhibition alters phagocyte recruitment, uptake, or bacterial killing in this setting. Airway organoid co-culture models that recapitulate epithelial architecture and polarity may also provide an intermediate platform to dissect these host-pathogen interactions in a more physiologic context. Additionally, while we demonstrate that microtubule stabilization plays a central role in reducing bacterial adherence and internalization, the precise downstream effectors—such as specific cell surface receptors, trafficking pathways, or polarity determinants—remain to be elucidated. Identifying these molecular targets will be essential for understanding the full mechanism of HDAC6-dependent protection. Further mechanistic studies are essential to delineate these pathways and to assess potential off-target or compensatory effects associated with HDAC6 inhibition. While the nocodazole treatment and TPPP cell data support the microtubule hypothesis, other potential targets and/or mechanisms could involve HDAC6 effects on innate immunity and/or inflammatory signaling that could be explored further.

In conclusion, our findings support HDAC6 as a key therapeutic target to disrupt early bacterial colonization in CF. Its inhibition offers a novel means of bolstering host defenses against PA by restoring microtubule stability and epithelial function, thereby limiting PA attachment and reducing the likelihood of progression to chronic infection. These findings provide a strong rationale for further investigation of HDAC6-targeted therapies as a complementary approach to existing CF therapeutics and antimicrobial treatments.

## MATERIALS AND METHODS

### Cell culture and treatment

Nasal epithelial tissue from wild-type (WT, C57BL/6J, B6), cystic fibrosis (CF, *Cftr^F508del^*^/*F508del*^), HDAC6 null (HDAC, *Hdac6*^−/−^), CF deficient of HDAC6 (CF/HDAC, *Cftr^F508del^*^/*F508del*^/*Hdac6*^−/−^), and TPPP null (TPP, *Tppp*^−/−^) mice was harvested and cultured at 37°C in T-75 flasks (Corning Inc., Corning, NY, USA) within a 95% O_2_–5% CO_2_ incubator (23, 31, 42). The cells were maintained in F12 medium supplemented with Y-27632, penicillin, and streptomycin. Primary HNE cells were similarly maintained and sourced from the cell culture core facility at the Case Western Reserve University (CWRU) CF Center and Lurie Children’s Hospital (IRB 2020-3807). Human BE cell lines, 16HBE14o^−^ (WT CFTR) and CFBE41o^−^ (F508del/F508del variants in CFTR), were obtained through Sigma-Aldrich. They were grown at 37°C in a 95% O_2_–5% CO_2_ incubator with minimal essential medium (Gibco, Carlsbad, CA, USA) supplemented with 10% fetal bovine serum containing 1 unit/mL penicillin and 1 µg/mL streptomycin. All cells were cultured and grown at the ALI in PneumaCult ALI medium (StemCell, Cambridge, MA, USA).

### Mice

Wild-type (WT, C57BL/6J, B6), cystic fibrosis (CF, *Cftr*^F508del/F508del^), HDAC6 null (HDAC, *Hdac6*^−/−^), CF deficient of HDAC6 (CF/HDAC6 KO), *Cftr*^F508del/F508del^/*Hdac6*^−/−^), and TPPP null (TPP, *Tppp*^−/−^) mice were cared for by the CF animal core facility at CWRU. All mice had unrestricted access to water and solid chow (Teklad 7904; Envigo). CF mice also received an osmotic laxative, PEG-3350 with electrolytes (Affordable Pharmaceuticals LLC), in their water to prevent intestinal blockage. The animals were housed in standard polysulfone microisolator cages with corncob bedding, in ventilated units, and maintained on a 12 h light/dark cycle at an average ambient temperature of 22°C.

### Pharmacologic treatments

WT and CF cells were subjected to treatment regimens with the HDAC6-selective inhibitor tubastatin (10 µM × 24 h) (28), microtubule disruptor nocodazole (40 µM × 30 min) (43), and/or vehicle (DMSO) prior to the assays outlined.

### Bacterial adherence and internalization assays

HNE, HBE, and MNE cells were seeded onto 24-well transwell filters (Corning, NY, USA) at a density of 100,000 cells per well in duplicate or triplicate for each biological replicate (minimum of three per assay). After a few days of expansion in submerged culture, the apical medium was removed to promote differentiation and polarization at ALI for a minimum of 21 days. Cells were monitored under the microscope to confirm confluency and the development of cilia. Mucus was routinely cleared from the apical surface of cells by washing with Dulbecco’s phosphate-buffered saline (D-PBS) three times, including just prior to all assays. Cells were infected apically with PA clinical CF isolate PA-C073A or laboratory strain PA14 at a multiplicity of infection of 1:30. Complementary experiments were performed with a donated and previously validated strain of PA lacking a functional type 3 secretion system, a *pscD* mutant (32). The actual bacterial inoculum was confirmed for each experiment. For adherence assays, cells were infected for 30 min, after which non-adherent bacteria were removed by gentle washing, and the cells were lysed for bacterial quantification via plating on LB agar. Bacterial adherence was quantified as the percentage of inoculum adhered. For internalization assays, cells were infected for 2 h, then treated with gentamicin (50 µg/mL for 2 h) post-infection to kill extracellular bacteria, followed by cell lysis and bacterial quantification as described above. Bacterial internalization was quantified as the percentage of adhered bacteria that were internalized to normalize for baseline differences in adherence.

### HDAC6 and TPP knockout experiments

Primary MNE cells were harvested from WT, CF, HDAC, CF/HDAC, and TPP mice (23). These cells were grown at ALI, and bacterial adherence and internalization assays were performed as outlined.

### Cell viability

MNE cells were seeded onto 24-well plates, grown at ALI, and then treated with DMSO, tubastatin, and/or nocodazole. Cells were infected apically with PA and steps performed as outlined for the bacterial adherence assay; however, at the end of the 30 min permitted for bacterial adhesion, cells were detached with trypsin for 5 min at 37°C. Equal volumes of cell suspension and trypan blue were combined, and 10 µL of the mixture was loaded onto Countess slides. The Invitrogen Countess 3 was used for quantifying viable vs non-viable cells.

### Western blotting

MNE cells were grown at ALI on transwell filters for a minimum of 21 days, then lysed with lysis buffer (50 mM Tris, pH 7.5, 1% Triton-X-100, 100 mM NaCl, 50 mM sodium fluoride, 250 µM sodium orthovanadate) or RIPA buffer (50 mM Tris HCl, pH 7.5, 150 mM NaCl, 1% Triton-X-100, 0.5% sodium deoxycholate, 0.1% SDS, 1 mM EDTA, 10 mM sodium fluoride) with 1 mM PMSF at 4°C for 30 min. Protein lysates (20 µg) were separated via SDS-PAGE on 10% polyacrylamide gels and transferred to PVDF membranes via Trans-Blot Turbo (BioRad) at 1 A and 25 V for 20 min. Membranes were blocked for 1.5 h at room temperature in 10% non-fat dehydrated milk in PBS with 0.1% Tween-20 (PBS-T), then incubated with primary antibodies, actin (Sigma A2066; 1:3,000 dilution) and acetylated α-tubulin (Santa Cruz SC23950; 1:1,000 dilution), at 4°C overnight. Membranes were washed three times with PBS-T and incubated for 1.5 h at room temperature with corresponding secondary antibodies conjugated to horseradish peroxidase (Abcam AB6789 and Sigma-Aldrich SAB3700842) at 1:3,000 dilution. Blots were visualized with SuperSignal West Pico PLUS chemiluminescent substrate (Thermo Scientific) and imaged with Syngene G:box. Quantification of protein expression was performed with Genetools (Synoptics Ltd).

### Confocal microscopy

HNE cells were grown at ALI for a minimum of 21 days, and PA-C073A bacteria were introduced per the bacterial adherence assay protocol above. After removing non-adherent bacteria, cells were washed with D-PBS, then fixed and permeabilized with ice-cold methanol overnight at −20 °C and dipped in ice-cold acetone for 1 min. Cells were blocked for 1 h at room temperature with StemCell blocking buffer (2% goat serum, 1% BSA, 0.1% cold fish skin gelatin, 0.1% Triton-X-100, 0.05% Tween-20, 0.05% sodium azide, in PBS) then incubated overnight at 4°C with primary antibodies in primary antibody dilution buffer (1% BSA, 0.1% cold fish skin gelatin, 0.05% sodium azide, in PBS). Primary antibodies included rabbit polyclonal pseudomonas antibody (Abcam AB68538, 1:1,000 dilution) and acetylated α-tubulin (Invitrogen 32-2700, 1:250 dilution). Cells were incubated with secondary antibodies, anti-mouse AlexaFluor 488 (Abcam AB150113) and anti-rabbit AlexaFluor 594 (Invitrogen A11072), at 1:1,000 dilution in 0.05% sodium azide in PBS for 2 h at room temperature in the dark. Cells were counterstained with 5 µg/mL of 4′,6-diamidino-2-phenylindole in PBS for 10 min in the dark. Membranes were excised and mounted with ProLong Diamond Antifade Mountant (Invitrogen) for imaging on a Zeiss LSM 880 confocal microscope with 40× Zeiss water immersion objective.

### Statistical analysis

Significance across bacterial adherence and internalization assays was determined by two-way ANOVA with *post hoc* Tukey’s multiple comparisons test. Significance across follow-up bacterial adherence assays with *pscD* mutant strain was determined by two-way ANOVA with planned comparisons. Statistical significance for western blots was determined by repeated-measures one-way ANOVA with Tukey’s multiple comparisons test.

## SUPPLEMENTAL MATERIAL

Supplemental tables iai.00225-25-s0001.pdf Tables S1 to S11.

ASM does not own the copyrights to Supplemental Material that may be linked to, or accessed through, an article. The authors have granted ASM a non-exclusive, world-wide license to publish the Supplemental Material files. Please contact the corresponding author directly for reuse.
