# Supplementary material for: HDAC6 inhibition reduces Pseudomonas aeruginosa adherence and internalization in cystic fibrosis epithelial cells via microtubule stabilization
Source: Infect Immun. 2026 Aug 12;94(9):e00225-25. doi: 10.1128/iai.00225-25 (PMC13552101; doi:10.1128/iai.00225-25)
Supplement: Supplementary file 1 [file iai.00225-25-s0001.pdf]

Table S1: Figure 1A: MNE @ ALI - PA-C073A

	WT MNE		CF MNE	
Condition	DMSO	Tubastatin	DMSO	Tubastatin
Mean Total Recovered CFU/well	8.95E+06	8.64E+06	1.06E+07	9.38E+06
Mean Adherent CFU/well	2.70E+06	2.13E+06	4.08E+06	2.73E+06

Table S2: Figure 1B: MNE @ ALI -PA14

	WT MNE		CF MNE	
Condition	DMSO	Tubastatin	DMSO	Tubastatin
Mean Total Recovered CFU/well	5.94E+06	6.33E+06	6.46E+06	6.27E+06
Mean Adherent CFU/well	1.92E+06	1.66E+06	2.65E+06	2.05E+06

Table S3: Figure 1C: HNE @ ALI -isogenic control vs. pscD mutant

	Isogenic Control		pscD mutant	
Condition	DMSO	Tubastatin	DMSO	Tubastatin
Mean Total Recovered CFU/well	4.55E+06	4.98E+06	3.83E+07	5.24E+07
Mean Adherent CFU/well	1.62E+05	8.58E+04	2.89E+06	3.42E+06

Table S4: Figure 1D: MNE @ ALI - PA-C073A Adherence

	WT MNE				CF MNE			
Condition	DMSO	Tubastatin	Nocodazole	Tubastatin + Nocodazole	DMSO	Tubastatin	Nocodazole	Tubastatin + Nocodazole
Mean Total Recovered CFU/well	6.20E+06	6.01E+06	6.45E+06	5.33E+06	6.72E+06	6.21E+06	6.64E+06	6.42E+06
Mean Adherent CFU/well	2.05E+06	1.53E+06	3.31E+06	2.13E+06	2.86E+06	1.98E+06	3.86E+06	2.93E+06

Table S5: Figure 2A: MNE @ ALI - PA-C073A Adherence

	MNE			
Condition	WT	HDAC6KO	CF	CF/HDAC6 DKO
Mean Total Recovered CFU/well	4.72E+06	4.82E+06	4.93E+06	4.80E+06
Mean Adherent CFU/well	1.49E+06	1.81E+06	2.04E+06	1.70E+06

Table S6: Figure 2B: MNE @ ALI - PA-C073A Adherence

	MNE		
Condition	WT	TPPP KO	CF
Mean Total Recovered CFU/well	4.93E+06	4.92E+06	5.67E+06
Mean Adherent CFU/well	1.68E+06	1.86E+06	2.43E+06

Table S7: Figure 3A: PA-C073A Internalization

	WT MNE		CF MNE	
Condition	DMSO	Tubastatin	DMSO	Tubastatin
Mean Total Recovered CFU/well	3.23E+06	2.70E+06	4.00E+06	3.00E+06
Internalized CFU/well	3.10E+05	1.79E+05	6.10E+05	3.54E+05

Table S8: Figure 3B: PA14 Internalization

	WT MNE		CF MNE	
Condition	DMSO	Tubastatin	DMSO	Tubastatin
Mean Total Recovered CFU/well	3.40E+06	2.60E+06	4.29E+06	3.58E+06
Internalized CFU/well	3.38E+05	1.98E+05	6.37E+05	4.37E+05

Table S9: Figure 3C: PA-C073A Internalization

	MNE			
Condition	WT	HDAC6KO	CF	CF/HDAC6 DKO
Mean Total Recovered CFU/well	3.14E+06	3.39E+06	3.88E+06	3.04E+06
Internalized CFU/well	2.75E+05	3.15E+05	5.58E+05	2.80E+05

Table S10: Figure 4A: PA-C073A Adherence

	16HBE14o-		CFBE41o-	
Condition	DMSO	Tubastatin	DMSO	Tubastatin
Mean Total Recovered CFU/well	6.61E+06	6.72E+06	6.75E+06	6.65E+06
Mean Adherent CFU/well	1.08E+06	8.27E+05	1.50E+06	9.78E+05

Table S11: Figure 4B: PA-C073A Adherence

	WT HNE		CF HNE	
Condition	DMSO	Tubastatin	DMSO	Tubastatin
Mean Total Recovered CFU/well	5.16E+06	5.59E+06	5.69E+06	5.87E+06
Mean Adherent CFU/well	8.47E+05	6.56E+05	1.33E+06	9.76E+05

Supplemental Tables S1-S11: Absolute bacterial recovery from adherence and internalization assays. Mean total recovered, adherent, or internalized *Pseudomonas aeruginosa* colony-forming units (CFU) per well are shown for the experiments presented in Figures 1-4. For each biological replicate, technical replicates were first averaged; the values presented here represent the means of all independent biological replicates. Tables include experiments performed with mouse nasal epithelial cells, primary human nasal epithelial cells, and human bronchial epithelial cell lines under the indicated pharmacologic and genetic conditions outlined in the manuscript. For adherence assays, total recovered CFU included bacteria recovered from the apical supernatant and epithelial cell-associated fractions. For internalization assays, internalized CFU were quantified following extracellular bacterial killing with gentamicin.

Abbreviations: ALI, air–liquid interface; **CF**, cystic fibrosis; **CFBE41o-**, cystic fibrosis bronchial epithelial cell line; **CFU**, colony-forming units; **DKO**, double knockout; **DMSO**, dimethyl sulfoxide**; HDAC6**, histone deacetylase 6; **HNE**, human nasal epithelial cells; **KO**, knockout; **MNE**, mouse nasal epithelial cells; **PA**, *Pseudomonas aeruginosa*; **TPPP**, tubulin polymerization-promoting protein; **WT**, wild type.
